# Consciousness in deep hypothermic circulatory arrest: a feasibility study

**DOI:** 10.1186/s13019-025-03484-w

**Published:** 2025-05-27

**Authors:** Joshua Ross, Thomas Jan, Deane Smith, Anelly Gonzales, Aubrey Galloway, Natalia Leontovich, Tara Keshavarz, Analise Dickinson, David Friedman, Emmeline Koopman, Elise Huppert, Ian Jaffe, Christopher Burke, John Kern, Paul Stelzer, Ashraf Sabe, Rebecca Spiegel, Andrew Klein, Arvind Rajagopal, Gage Parr, Charles Deakin, Sam Parnia

**Affiliations:** 1 NYU Langone Health, 550 1 stAvenue, New York, NY 10010 USA; 2 University of Washington, 1959 NE Pacific St, Seattle, WA 98195 USA; 3 Univeristy of Virginia Health, 1215 Lee St, Charlottesville, VA 22903 USA; 4 Mount Sinai Hospital, 1468 Madison Ave, New York, NY 10029 USA; 5 Brigham and Women’s Hospital, 75 Francis Street, Boston, MA 02115 USA; 6 Stony Brook University Hospital, 101 Nicholls Road, Stony Brook, NY 11794 USA; 7 Royal Papsworth Hospital, Papworth Road, Cambridge, UK; 8 Rush University Medical Center, 1620 W Harrison St, Chicago, IL 60612 USA; 9 George Washington University Hospital, 900 23rd Street NW, Washington, DC 20037 USA; 10 Southampton General Hospital, Tremona Road, Southampton, UK

**Keywords:** DHCA, Cardiac Arrest, Circulatory arrest, Consciousness, Learning, EEG, Cardiac standstill

## Abstract

**Background:**

Studies have not explored consciousness during deep hypothermic circulatory arrest (DHCA). However, as studies in cardiac arrest have identified a spectrum of consciousness, we sought to establish the feasibility of studying consciousness during DHCA.

**Methods:**

This was a prospective study across 10 hospitals with 36 DHCA patients undergoing thoracic aortic aneurysm repair or pulmonary endarterectomy. A tablet computer delivered audiovisual stimuli (images and names of three fruits) using headphones during each procedure as a potential test of implicit learning and explicit recall. We also established electroencephalography (EEG) and near-infrared spectroscopy (NIRS) to measure electrocortical markers of consciousness and cerebral oxygenation. Post-procedure interviews were carried out to test patients’ ability to recall the audiovisual stimuli as well other explicit memories. Primary outcomes: 1) Feasibility of establishing tests of explicit recall and implicit learning, 2) Electroencephalography testing during DHCA. Secondary outcomes: 1) Signs of explicit recall of memories or implicit learning, and 2) identification of electrocortical biomarkers of consciousness during DHCA.

**Results:**

Overall, 29/36 (81%) had the tablet set up. All 36 had NIRS and EEG set up, but 9 (25%) had useable EEG data, and 23 (66%) NIRS data. Delta EEG waves were observed during circulatory arrest in 3/9 (33%) patients, while 1/9 (11%) had theta waves just prior to circulatory arrest. All others showed isoelectric pattern. 35/36 (97%) agreed to follow up interviews. None had explicit recall of the names of the three fruits, but 3/36 (9%) correctly guessed them as a potential sign of implicit learning and 3 (9%) recalled other memories including events around the procedure and themes consistent with a recalled experience of death.

**Conclusions:**

A spectrum of consciousness and awareness, including signs of implicit learning and electrocortical biomarkers of consciousness may be present during DHCA, despite absence of visible signs of consciousness. This can be further used to help explain the negative psychological outcomes that cardiac arrest survivors face.

**Supplementary Information:**

The online version contains supplementary material available at 10.1186/s13019-025-03484-w.

## Introduction

Roughly 350,000–750,000 cardiac arrests occur each year in the United States, with ~ 10% survival rate [1]. Several studies report negative psychological and cognitive outcomes associated with survivorship after cardiac arrest, including anxiety (13–41%), depression (15–45%), and post-traumatic stress disorder (PTSD) (20–50%) while others report positive outcomes including a transcendent recalled experience of death (RED) (9–21%) including reviewing life events, a sense of being transcendent, and a sense of being home [2–5].

Despite interest by the American Heart Association, cardiac arrest related cognitive activity including awareness and consciousness remain poorly understood [6, 7]. Although there is an assumption of consciousness being absent in cardiac arrest, the AWAreness during REsuscitation (AWARE) multisite study of 2060 in-hospital cardiac arrest patients, of which 140 survived, showed that 39% reported a perception of awareness without any explicit recall of events, while 9% had cognitive experiences compatible with a RED. A further 2% described explicit audiovisual recall of the cardiac arrest events. This supported similar findings from a prior multisite study of 344 in-hospital cardiac arrest, in which ~ 20% of the survivors reported experiences compatible with a RED, including visual awareness [8].

In the more recent, AWARE-II study of 567 cardiac arrest patients, of which 53 survived, a perception of awareness without explicit recall was reported by 39% of survivors. Overall, 21% had experiences compatible with a RED and 2% recalled explicit auditory and visual awareness [9]. Aside from explicit awareness, consciousness may take the form of implicit learning, which has previously been described in patients under general anaesthesia [10–12]. Thus, AWARE-II also incorporated tests of implicit learning, as well as electroencephalography (EEG) during CPR to detect electrocortical biomarkers of consciousness during cardiac arrest/standstill, especially as electrocortical brain waves consistent with lucid consciousness during and after cardiac standstill and death have been described [13–16].

Still, it is unclear if conscious awareness – whether explicit or implicit—may contribute to psychologic outcomes, such as PTSD, while the RED may contribute to life enhancing or positive outcomes including becoming less materialistic and less afraid of death [2]. Considering the complexity of consciousness during cardiac standstill which may contribute to longer-term psychological outcomes, including anxiety, depression, and PTSD, it is imperative to study this systematically. However, low survival rates make this challenging during active cardiopulmonary resuscitation in cardiac arrest, necessitating the use of alternative models [13].

Deep hypothermic circulatory arrest (DHCA) is a state of cardiac standstill/arrest due to induced hypothermia at temperatures less than 20 °C to allow for temporary cessation of blood flow and is used in cardiothoracic and neurosurgical settings. Due to the physiological similarities between DHCA and cardiac arrest, DHCA can serve as a model for researching consciousness during cardiac standstill states [17]. Although as with cardiac arrest, there is an assumption of the absence of consciousness, a retrospective questionnaire-based study among 70 people identified mental activity, including self-reported episodes of visual-awareness and the ability to describe the operating room staff, environment, and seeing bright lights [18].

Currently, there are no prospective studies of consciousness during DHCA, which we hypothesize may be present in some form, similar to cardiac arrest. We therefore set out to assess the feasibility of incorporating tests of consciousness, by building on the methods developed during the AWARE-II study—including tests of implicit learning and explicit recall as well as EEG and cerebral oximetry—in patients undergoing DHCA to enable a larger future study of consciousness during DHCA.

## Methods

### Study population and enrollment

This was a prospective feasibility study amongst patients receiving DHCA for either thoracic aortic aneurysm repair or pulmonary endarterectomy to develop and optimize study methods. The protocol received ethics committee approval from all sites prior to enrolling the first participant at each site. Patients were followed until discharge.

Eligible patients undergoing elective DHCA procedures were identified from outpatient clinics and informed consent obtained. Participants were recruited between 7/2020 and 1/2022 and enrolled from six different sites: Royal Papworth Hospital (Cambridge, UK), University of Virginia Medical Center (Charlottesville, VA, USA), George Washington University Medical Center (Washington, D.C, USA), Brigham and Women’s Hospital (Boston, MA, USA), NYU Tisch Hospital (New York City, NY, USA), and Rush University Medical Center (Chicago, IL, USA). Inclusion criteria were patients 18 years old or older and undergoing a DHCA procedure. Exclusion criteria were patients with underlying neurocognitive defects.

### Establishing tests of visual and auditory awareness during DHCA

To test for hidden consciousness, a tablet and headphones were utilized during the circulatory arrest portion of DHCA with audiovisual stimuli as described in the AWARE-II study [9]. Research staff were trained in positioning and use of tablet and headphones and headphone audio was tested with another application prior to placement on patient. After placing the tablet and securing headphones, a preinstalled tablet application developed for AWARE-II was started during the beginning of circulatory arrest. The tablet application randomly displayed one of ten predetermined images (Figure S1) and played a particular audio file with Bluetooth enabled headphones consisting of three fruits (“Apple, Pear, Banana”) while recording the time of each cue in order to assess for potential explicit recall and implicit learning. Audio cues were programmed to start five minutes after the commencement of circulatory arrest and repeated every five minutes for up to 40 min or until the procedure finished. Standard protocols per the clinical sites were otherwise followed regarding sterility, cooling and protection of the head and eyes.

### Real-time cerebral oximetry and electroencephalography during DHCA

Cerebral oximetry was incorporated as a noninvasive marker of cerebral oxygenation. This uses Near-Infrared Spectroscopy (NIRS) to transmit and detect near-infrared light through forehead sensors. One adhesive rSO_2_ sensor was applied to the lateral forehead containing two light sources and detectors, one penetrating ~ 3 cm into the frontal region of the brain reflecting venous hemoglobin saturation and one collecting absorption from superficial tissue subtracted from overall measurements [19]. Using an internal algorithm, devices calculated rSO_2_ using the unique absorption spectra of oxy- and deoxyhemoglobin (detection range 0–100%, normal 60–80%).

Electroencephalography was used as a noninvasive measure of electrocortical function in during cardiac standstill and DHCA. Prior to study use, EEG equipment was tested on healthy controls. The EEG sensors, which comprised two temporal, two frontal, and two midline grounding electrodes attached to an adhesive strip were placed across the forehead.

Prior to the start of the procedure, the research tablet was paired with headphones and placed on the patient along with the cerebral oximetry device (Equanox, Nonin, USA) and portable EEG (SedLine Masimo, Irvine, CA, USA). Continuous data for both devices were collected and event marked throughout the surgery, along with continuous temperature monitoring with time points recorded during the cooling phase (35 °C, 32 °C, and 25 °C) and the warming phase (32 °C and 36 °C).

EEG was interpreted by one board-certified neurophysiologist (D.F.) blinded to outcome using Persyst 14 software (Salona Beach, CA, USA). EEG and rSO_2_ values were matched by timestamp and examined from five minutes before circulatory arrest to five minutes after. The interpretable EEG images were further classified as “near normal” (alpha, beta, theta, delta activity) or “absent” (suppression).

A file was created for each subject and the data was stored under the subject’s unique research identification number in RedCap (Version 13.1.29, Vanderbilt University, Nashville, TN, USA). Data regarding the use of each device, including the transfer of data was concurrently collected and stored.

### Analysis of survivors’ explicit recall, implicit learning, and cognitive recollection

After the procedure, patients were followed daily and if sufficiently recovered, including from a neurocognitive standpoint (determined using a standardized validated Abbreviated Mental Test (AMT) > 8), were offered the opportunity to complete a 45-min interview to assess consciousness, including implicit learning and explicit recall. During this interview, research staff examined explicit recall first by asking what images may have been seen on the tablet or words heard from the headphones, followed by showing the bank of 10 images in a random order and asking if any were recalled. Implicit learning was tested by judging preferences for images and fruits presented during the procedure (“Select the image from this bank of 10 images that you prefer”, “Randomly pick the first three fruits that come to your mind when thinking about your time unconscious”).

Subjects were also interviewed about events and details that they were able to recall from the procedure. These interview questions were constructed according to the standard cognitive interview technique by Geiselman used to retrieve information from a memory, shown in further detail in supplemental material^13^. Subjects were then administered a survey (Greyson Scale) to quantify experiences consistent with elements of RED based on cognitive, perceptual, and emotional experiences.

### Data analysis & statistics

Data was compiled and summarized from RedCap. Summaries of scripted interviews were reviewed quantitatively and results were grouped based upon themes. The correlation between brain activity waveforms and cerebral oxygen levels was explored through Spearman’s rho in subjects with data available. Data was analyzed with SAS 9.3 (SAS Institute Inc., Cary, NC). Friedman test was used to measure significance between cerebral oximetry during the different temperature points.

## Results

A total of 56 patients were initially screened undergoing a DHCA procedure. Of those, 36 patients were recruited into this study, with demographics and patient information summarized in Table 1.

**Table 1:** Enrolled patient demographics and descriptive DHCA procedure statistics

	**Overall**
N	36
Age (mean(SD))	59.78 (15.78)
Sex = Male (%)	23 (63.9)
Race (%)	
White	30 (83.3)
African Descent	3 (8.3)
Other	1 (2.8)
Unknown	2 (5.6)
Total Cardiopulmonary Bypass Time in hours (mean (SD))	4.66 (1.21)
Core Temperature before the start of the procedure (mean (SD))(Degree Celsius)	36.37 (0.37)
Lowest Temperature Achieved during the Procedure (mean (SD))(Degree Celsius)	19.70 (2.87)
Site (%)	
Royal Papworth Hospital	25 (69.4)
University of Virginia	4 (11.1)
George Washington University	2 (5.56)
Brigham and Women's	2 (5.56)
New York University	2 (5.56)
Rush University	1 (2.78)

### Feasibility of incorporating a tablet for test of consciousness

Overall, in 29/36 (86%) the tablet was used successfully. The reasons for the inability to use the tablet in others, as shown in Table 2, included files not uploading to RedCap, application prematurely closing, and touchscreen not working.

**Table 2:** Reasons for tablet failure during DHCA

**Tablet Failure Reason**	N (%)
No file uploaded to RedCap	3 (8.3)
Application design failure^a^	1 (2.8)
Application prematurely closing	1 (2.8)
Tablet failed to display images	1 (2.8)
Tablet touchscreen failure	1 (2.8)

^a^Application was built to run during a single circulatory arrest. One procedure included multiple circulatory arrests

### Post-operative interviews to assess implicit learning and explicit recall

Thirty-five of the 36 (97.2%) of patients were interviewed after DHCA. Interviews were conducted median 6 days after surgical procedure while in hospital. None of the patients had explicit recall of visual or auditory stimuli (seeing image on the tablet, hearing words from headphones). However, when asked to randomly pick three fruits that were played during DHCA, 3 of 35 (8.6%) were correctly able to do so, suggestive of possible implicit learning.

Three of the 35 patients (8.6%) had recalled memories in relation to the overall procedure, as outlined in Table 3, such as hearing conversations and recalling blood draw attempts. One of these patients had an experience consistent with a possible recalled experience of death.

**Table 3:** Research team recordings of qualitative patient DHCA procedure during post procedure interview

Patient A	“Remembers a conversation with God. Whether to go to the bad place or good place.”
Patient B	“The patient recalls being annoyed by Australian voices shouting. And after the procedure, he recalls a nurse tried to take blood from his left wrist 3 times but was unable to. When he approached the nurse this one stated that she did not try to draw blood from him.”
Patient C	“Like Ground hog day kept saying same things over and over. Didn't think it was going to stop saying the name of another doctor. Felt like the same person saying it forever. Freaked me out and"Lots of laughing". Patient points to outside of ICU Room"out there". Mentions the rooms kept changing.”

### Tests of cerebral electrical activity

Overall, 9 of 36 patients (25%) completed EEG. The reasons for the inability to complete EEG in 27 patients are shown in Table 4, including failure of files to transfer to RedCap, lead placement adherence issues, and event time mismatching, which were addressed with further staff training.

**Table 4:** EEG Equipment errors broken down by reason

**EEG Equipment**	N (%)
Usable File	9 (25)
Unusable File	27 (75)
EEG not reading accurately	1 (2.8)
Expired sensors	1 (2.8)
No file transferred to RedCap	17 (44.7)
Start/End time mismatch	2 (5.9)
High impedance errors	1 (2.8)
No brain signal	1 (2.8)
Poor sensor adherence	3 (8.8)
Leads not attached properly	1 (2.8)

In the 5-min period before circulation had stopped, 1 patient (11.1%) showed theta waves, while the remaining 8 patients demonstrated isoelectric activity. During the period of circulation cessation, 3 of 9 patients (33.3%) demonstrated delta waves. In the period when circulation resumed, brainwave activity remained absent in all. These findings are summarized in Table 5. One of the three patients who correctly chose the three-fruit played on the headphones showed delta wave activity during circulation pause (RPH-10019). The others did not have EEG data available. None of the three patients with recalled memories in relation to the procedure had EEG data available.

**Table 5:** Brain waves from nine DHCA patient EEG readings before, during, and after circulatory arrest

Patient ID	Brain waves in 5 min before Circulation paused	Brain waves during circulation pause	Brain waves in 5 min after circulation resumption
RPH-10010	Isoelectric (Suppression)	Delta wave	Isoelectric (Suppression)
RPH-10019	Isoelectric (Suppression) + Artifact	Delta wave	Isoelectric (Suppression)
RPH-10021	Isoelectric (Suppression)	Isoelectric (Suppression)	Isoelectric (Suppression)
RPH-10022	Theta wave	Isoelectric (Suppression)	Isoelectric (Suppression)
RPH-10026	Isoelectric (Suppression)	Isoelectric (Suppression) + artifact	Isoelectric (Suppression)
RPH-10030	Isoelectric (Suppression)	Isoelectric (Suppression)	Isoelectric (Suppression)
RPH-10032	Isoelectric (Suppression)	Isoelectric (Suppression)	Isoelectric (Suppression)
RPH-10033	Isoelectric (Suppression) + artifact	Isoelectric (Suppression) + artifact	Isoelectric (Suppression) + artifact
RPH-10034	Isoelectric (Suppression)	Delta wave	Isoelectric (Suppression)

### Tests of cerebral oximetry

Twenty-three of the 36 patients (65.7%) had successful cerebral oximetry data collection. In the remaining patients, errors included a failure to generate data files and date files not being correctly transferred from clinical sites, as further detailed in Table 6. These were resolved by training.

**Table 6:** Brain oximetry equipment errors

**Brain Oximetry Files**	N (%)
Usable	23 (65.7)
Unusable	13 (34.3)
Wrong monitor used	1 (2.8)
File not uploaded	3 (8.4)
< 2 min of data	1 (2.8)
No file generated	6 (17.1)
Non-continuous monitoring	1 (2.8)
Unreadable file	1 (2.8)

Temperature was collected at all time points for 21 of the 36 patients (58.3%). Overall data available regarding the EEG, cerebral oximetry, and temperature data collection is detailed in Table 7.

**Table 7:** Brain function monitoring data availability

**Brain Function Data Available**	N (%)
Brain Oximetry & Temperature	15 (41.7)
Brain Oximetry & EEG	2 (5.56)
EEG & Temperature	6 (16.7)
Brain Oximetry & EEG & Temperature	2 (5.56)

**Fig. 1:**
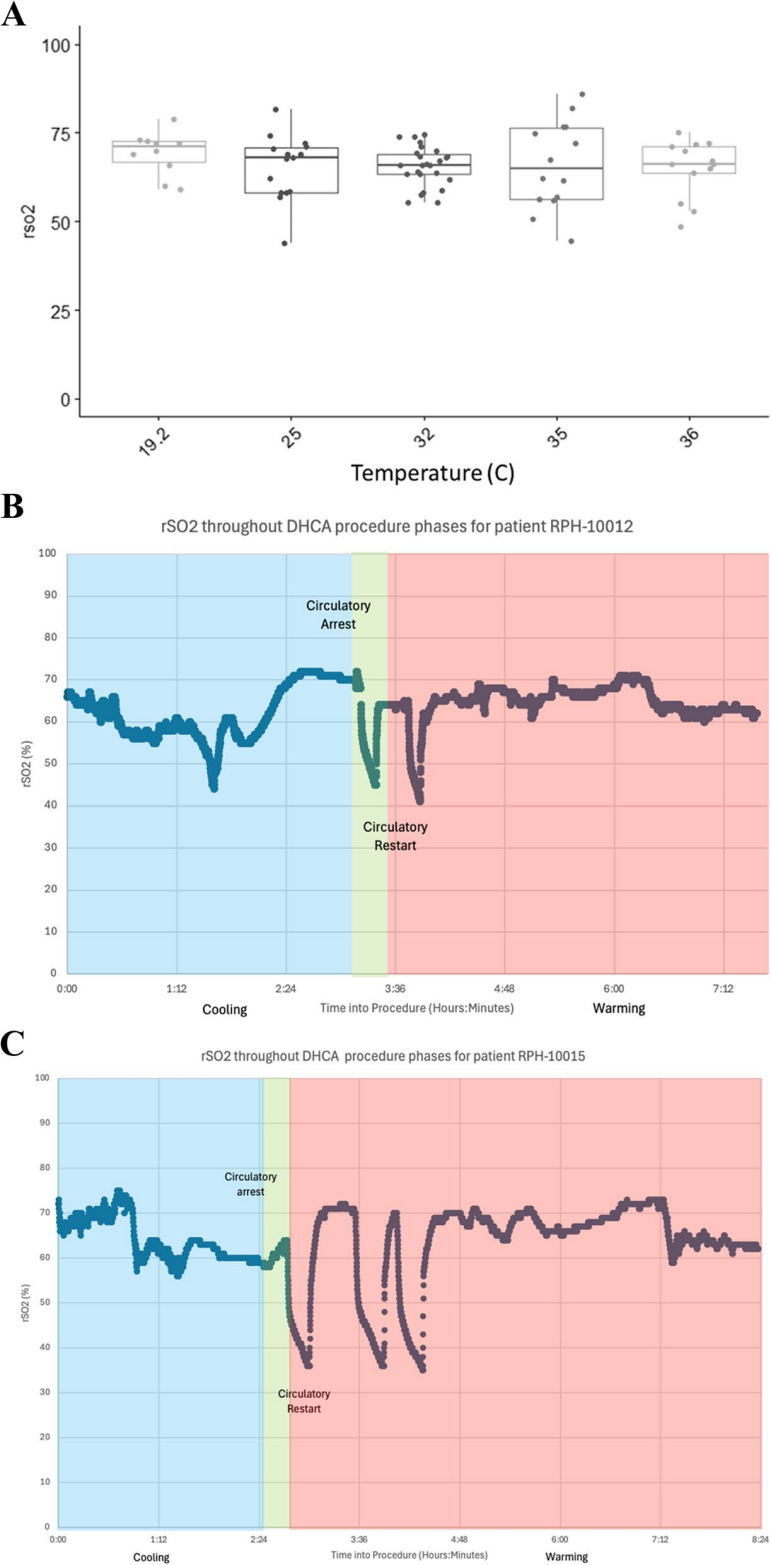
**A** Graph of median rSO2 collected at different temperature (C) time points of DHCA. No statistical difference was noted between rSO2 at different temperatures while circulation was present. **B** Brain oximetry throughout DHCA procedure phases for patient RPH-10012. Precipitous drops in cerebral oximetry are noted immediately following circulatory arrest. **C** Brain oximetry throughout DHCA procedure phases for patient RPH-10015

In the 15 patients with all temperature time points collected and brain oximetry data, the results were compiled in Fig. 1A. Median rSO2 was not significantly different at any recorded temperature point (median rSO2 74% at coolest temperature, median rSO2 72% at highest temperature). Figures 1B and C show individual rSO2 curves against the different time points of temperature and the DHCA procedure for two patients, noting precipitous drops in cerebral oximetry following circulatory arrest subsequent increases upon restarting of circulation.

## Discussion

This study demonstrated a level of feasibility in using brain monitoring during DHCA, capturing 25% of EEG data and 65.7% of cerebral oximetry data. While data collection was achieved, there is room for improvement. Moving forward with a larger study, we recognize a high degree of training and improved standardization are essential to ensure enhanced data collection. Despite not being able to obtain all data, we identified reports of possible implicit learning and cognitive recollections in 9% of patients, which is intriguing. We additionally demonstrated physiological EEG activity consistent with what is seen in people while asleep in 33% of patients undergoing DHCA while their cerebral oximetry it markedly below normal. Altogether, these data point to some potential level of consciousness during DHCA.

While these outcomes have not been looked at before with DHCA, studies have shown that after cardiac surgery, up to 20% of patients may meet criteria for major depressive disorder and ~ 14% develop new PTSD six months after surgery [20]. Similarly, after cardiac arrest, survivors may have poor psychological outcomes, with rates of anxiety, depression, and PTSD up to 61%, 45%, and 27% respectively [3]. While it had been assumed that patients undergoing circulatory arrest, whether it be through cardiac arrest or otherwise, had no consciousness, this notion may no longer hold true. This may provide a potential explanation for the long-term psychological outcomes seen after cardiac standstill.

Our study supports the single other study which examined consciousness in DHCA by Beauregard et al., which found that ~ 9% of patients undergoing DHCA had cognitive recollection from retrospective questionaries [18]. We also examined consciousness by testing implicit learning, with several patients exhibiting this for the first time in states of cardiac standstill, similar to prior anesthesia studies which found the incidence to be between 2%—12% [21, 22]. Given the small sample size as a feasibility study and inability to ensure adequate scrutiny of the recall process, it is unclear whether the recollection of fruits was due to chance from to the commonality of the three fruits rather than implicit learning. This may be clarified in further, more powered phases of this study. More importantly, we build upon already established work describing ‘recalled experiences surrounding death’ (RED) in patients interviewed after surviving a cardiac arrest, with consistent general themes of recollections that these patients experienced centering around ideas of a higher being and possible memories of the actual procedure. With that being said, the temporality of RED (experiences occurring during circulatory arrest versus before or after procedure) is inherently difficult to discern [23]. Finally, we confirm work done by our group measuring electrophysiologic markers of consciousness in states of cardiac standstill, which found EEG and brain oximetry markers of consciousness while undergoing CPR, including delta waves as also seen here in this study [24–27].

Taken together, these all suggest that some patients may exhibit a level of consciousness while seemingly unconscious during states of cardiac standstill. However, the mechanism of establishing consciousness is yet to be explored.

As a feasibility study, this study was limited in both data collection and study participants. Technical difficulties limited both device usage and data capture rate which will be addressed in further phases with improved staff training and standardization as well as and newer devices to allow for EEG and brain oximetry measurements from areas of the brain other than the frontal cortex. Larger studies are needed to determine whether signs of consciousness—either recalled or based on electrophysiological activity – impacts post-cardiac arrest/standstill psychological outcomes.

## Conclusion

In patients undergoing DHCA, there may be a spectrum of consciousness and awareness as suggested by possible implicit learning and explicit recall of events together with electrophysiological biomarkers during DHCA. These findings may have implications for long-term psychological outcomes following cardiac arrest/standstill states.

## Supplementary Information

Supplementary Material 1.

## Data Availability

No datasets were generated or analysed during the current study.
