# Supplementary material for: Consciousness in deep hypothermic circulatory arrest: a feasibility study
Source: J Cardiothorac Surg. 2025 May 27;20:242. doi: 10.1186/s13019-025-03484-w (PMC12117760; doi:10.1186/s13019-025-03484-w)
Supplement: Supplementary file 1 — Supplementary Material 1. [file 13019_2025_3484_MOESM1_ESM.pdf]

Supplemental Materials:

Figure S1: Image bank of randomly displayed image on tablet during DHCA procedure

<table>
<tr><td colspan="4">UK PICTURES
11.3 One image from the images below was present in the room while you were unconscious. We would be grateful if you could take a guess as to which one of the images below was in the room?</td></tr>
<tr><td>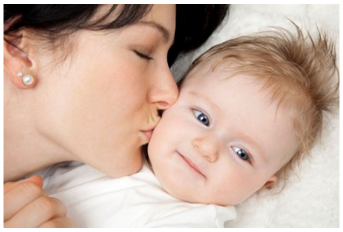</td><td>11.3.1
☐ no ☐ yes</td><td>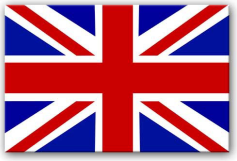</td><td>11.3.2
☐ no ☐ yes</td></tr>
<tr><td>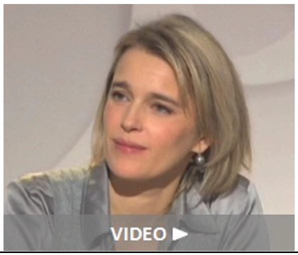</td><td>11.3.3
☐ no ☐ yes</td><td>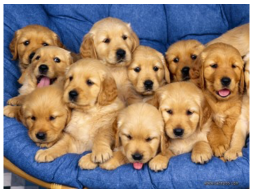</td><td>11.3.4
☐ no ☐ yes</td></tr>
<tr><td>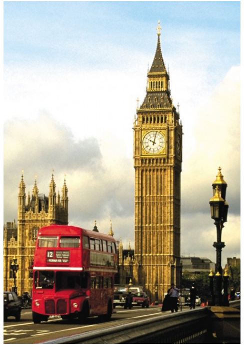</td><td>11.3.5
☐ no ☐ yes</td><td>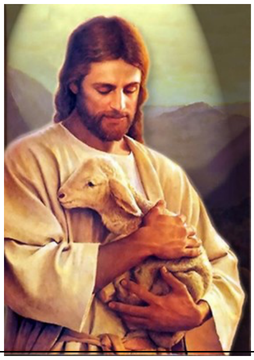</td><td>11.3.6
☐ no ☐ yes</td></tr>
<tr><td>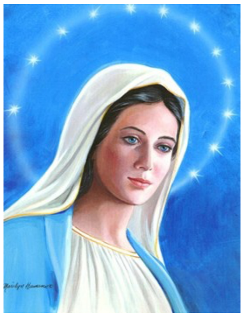</td><td>11.3.7
☐ no ☐ yes</td><td>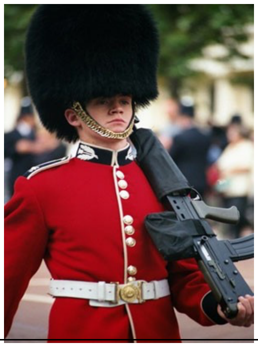</td><td>11.3.8
☐ no ☐ yes</td></tr>
</table>

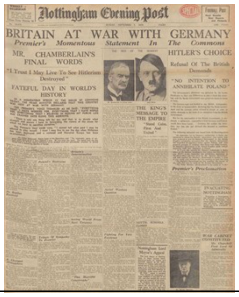

Nottingham Evening Post

BRITAIN AT WAR WITH GERMANY

Premier's Momentous Statement In The Commons

MR. CHAMBERLAIN'S FINAL WORDS

"I Trust I May Live To See Hitlerism Destroyed"

FATEFUL DAY IN WORLD'S HISTORY

HITLER'S CHOICE

Refusal Of The British Demands

"NO INTENTION TO ANNIHILATE POLAND"

THE KING'S MESSAGE TO THE EMPIRE

"Stand Calm, Firm And United."

Premier's Proclamation

EVACUATING NOTTINGHAM

11.3.9

☐ no ☐ yes

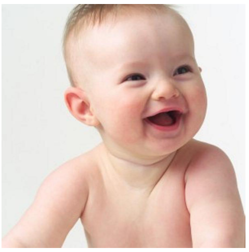

11.3.10

☐ no ☐ yes

US PICTURES

11.3 One image from the images below was present in the room while you were unconscious. We would be grateful if you could take a guess as to which one of the images below was in the room?

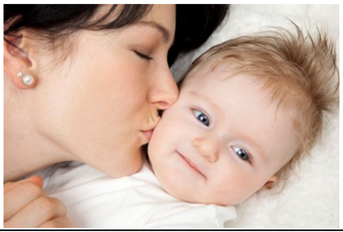

11.3.1

☐ no ☐ yes

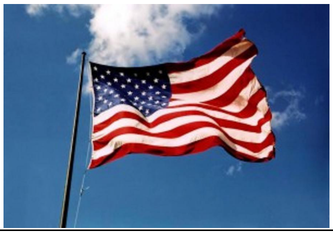

11.3.2

☐ yes

☐ no

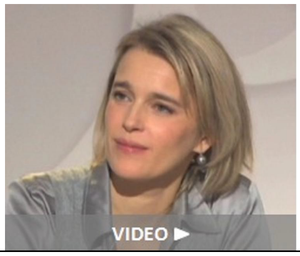

11.3.3

☐ no ☐ yes

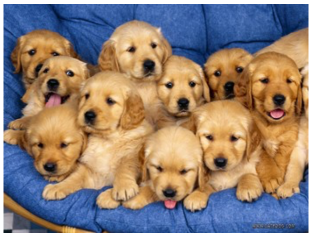

11.3.4

☐ yes

☐ no

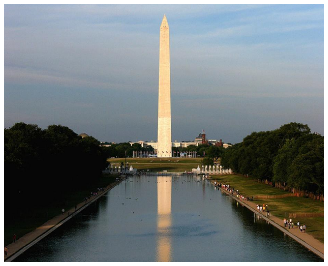

11.3.5

☐ no ☐ yes

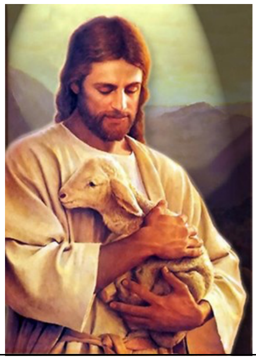

11.3.6

☐ yes

☐ no

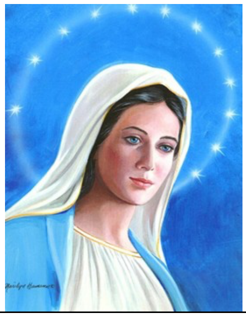

11.3.7

☐ no ☐ yes

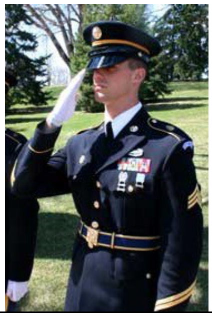

11.3.8

☐ yes

☐ no

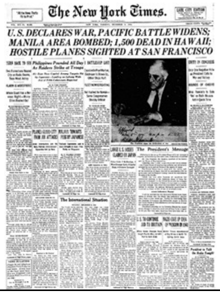
The New York Times.

U. S. DECLARES WAR, PACIFIC BATTLE WIDENS; MANILA AREA BOMBED; 1,500 DEAD IN HAWAII; HOSTILE PLANES SIGHTED AT SAN FRANCISCO

11.3.9

☐ no ☐ yes

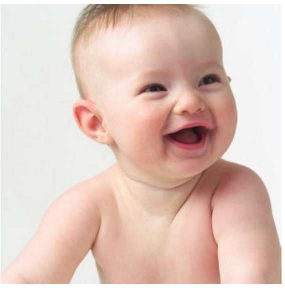

11.3.10

☐ yes

☐ no
